# Substitution of Wheat Flour with Modified Highland Barley Flour Affects Properties and Quality of Wheat Flour, Dough, and Noodles

**DOI:** 10.3390/foods15172958

**Published:** 2026-08-23

**Authors:** Mengdi Song, Shihong Wang, Zhan Liang, Huixian Wang, Jingshu Wang, Jihong Huang, Jianyong Song, Jie Zeng, Haiyan Gao

**Affiliations:** 1 School of Food Science, Henan Institute of Science and Technology, Xinxiang 453003, China; 15737319096@163.com (M.S.); 15203807430@163.com (S.W.); 15737053280@163.com (Z.L.); 16627686510@163.com (H.W.); 13782529904@163.com (J.W.); zengjie623@163.com (J.Z.); 2 Henan Bainong Seed Industry Co., Ltd., Xinxiang 453003, China; 3 Agricultural College, Henan University, Zhengzhou 475001, China; huangjih1216@126.com; 4 Henan Yingjie Food Co., Ltd., Xinxiang 453100, China; henanyingjieshipin@163.com

**Keywords:** superfine grinding, ultrasonic, highland barley flour, wheat dough, noodle quality

## Abstract

Highland barley (HB) is nutritionally rich but its low gluten content limits its use in wheat-based staple products. This study systematically compared the effects of substituting wheat flour with superfine grinding modified highland barley flour (SG-HBF) or ultrasonically modified highland barley flour (US-HBF) at 10–30% ratios on the properties and quality of wheat flour, dough, and noodles. Results showed that SG-HBF reduced the peak viscosity, breakdown, and setback value of the blended flour, enhanced its thermal stability and anti-aging properties; whereas, US-HBF significantly increased the viscosity. Noodles maintained good sensory and cooking quality when SG-HBF ≤ 15% or US-HBF ≤ 20%. Beyond these thresholds, the cooking loss increased sharply and overall acceptability declined. At the same substitution ratio, SG-HBF outperformed US-HBF in terms of water distribution, cooking loss, and sensory scores, offering better processing efficiency, while US-HBF provides higher springiness and lower broken rate, suitable for products requiring noodle integrity. This study provides a reference for the application of modified HBF in wheat-based products.

## 1. Introduction

In recent years, with the economic development and improvement of living standards, dietary adjustments have become one of the most effective methods for preventing and treating chronic diseases. People’s staple foods have shifted from refined flour to coarse grains such as corn, oats, buckwheat, and highland barley (HB). HB is used as food in many Asian countries, including Pakistan, Afghanistan, Japan, Nepal and China. Compared with many cereal crops, highland barley has unique chemical components, such as high β-glucan and dietary fiber content [1]. These components are the main reason for its antioxidant, anticancer and antibacterial properties [2]. Kumari et al. added highland barley flour (HBF) to wheat flour to prepare spaghetti, and found that the spaghetti had higher nutritional value and better cooking quality [3]. Al-Ansi et al. found that the germinated highland barley significantly improved the nutritional value, antibacterial and antioxidant properties of bread [4]. However, the higher dietary fiber and lower gluten protein content in HBF negatively affect the taste and processing quality of HB products [5]. Moreover, due to its low protein content, HBF cannot be processed into staple foods, such as bread, steamed bread, noodles and dumplings, which seriously limits its wide application in the food industry.

Many scholars have adopted various modification methods to improve the quality of HB products [6,7]. Physical modification methods are widely used due to their advantages of being green, safe, highly efficient, and easy to operate. Ma et al. found that superheated steam treatment increased the degree of gelatinization and water absorption index of wheat flour [8]. Zheng et al. studied the effects of pearling on the nutritional value of HBF and the processing characteristics of noodles. The results showed that this treatment changed the structure of HBF and improved the dough’s pasting viscosity and thermal mechanical properties [9]. Superfine grinding (SG) and ultrasonic (US) treatment are also common physical modification methods. SG mainly uses mechanical force or air flow to impact the starch particles, thereby destroying the crystal structure of the starch and reducing particle size. The US treatment mainly generates turbulence and shear forces through cavitation, disrupting starch crystals and altering their physicochemical and functional properties [10,11,12]. Liu et al. found that SG significantly affected wheat-barley dough properties and bread quality by weakening the gluten network [13]. Li et al. found that US treatment combined with epigallocatechin addition could modulate the physicochemical and digestibility properties of HB starch [14]. Although previous studies have investigated the effects of SG or US treatment on the physicochemical properties of HBF or its components, a systematic comparison of these physical modification methods in the context of wheat-based noodle production has not been reported. Most existing studies have focused on a single modification technology without addressing the differential impacts of SG and US on the processing characteristics of blended flour, dough, and final noodle quality. Furthermore, the specific advantages and limitations of each modification method for noodle applications remain unclear.

Therefore, this study systematically compared and analyzed the influence of different substitution ratios of SG-HBF or US-HBF on the quality of wheat flour, dough and noodles. The results are expected to provide valuable guidance for selecting appropriate modification strategies in the development of highland barley-enriched wheat products.

## 2. Materials and Methods

### 2.1. Materials

Wheat flour (weak gluten flour) was obtained from Xinxiang Embryo Grain Health Food Co., Ltd., Xinxiang, China. Highland barley flour was provided by Qinghai Qingzang Tribe Agriculture and Animal Husbandry Development Co., Ltd., Qinghai, China.

### 2.2. Preparation of Modified Highland Barley Flour

SG-HBF was prepared according to the method described by Liu et al. with slight modifications [15]. HBF was processed using a superfine grinding machine (KCW-701S, Beijing Kunjie Yucheng Machinery Equipment Co., Ltd., Beijing, China.) at a frequency of 30 Hz, followed by sieving through a 100 mesh sieve.

US-HBF was prepared according to the method described by Panghal et al. with slight modifications [16]. The HBF suspension (1:10, *w*/*v*, 100 mL) was made by adding HBF in a beaker containing water, and then was subjected to ultrasonication with a sonicator (JY 92-11DN, Ningbo xin-zhi biotechnology, Ltd., Ningbo, China, probe diameter 10 mm) for 60 min at frequency of 20 kHz. The glass beaker containing solution was maintained at 25 °C in an ice bath to avoid excess heating. The probe with diameter 10 mm was dipped into the solution at 10 mm depth with output power of 300 W in a pulse mode with 5 s on and 5 s off cycles. The slurry was centrifuged and dried in an oven at 40 °C for 48 h, then ground in pestle and mortar, followed by sieving through a 100 mesh sieve.

### 2.3. Preparation of Mixed Flour

Wheat flour was replaced with 10%, 15%, 20%, 25%, and 30% (*w*/*w*) of SG-HBF or US-HBF, respectively, and the mixed flours were named SG-HBF-10, SG-HBF-15, SG-HBF-20, SG-HBF-25 and SG-HBF-30 and US-HBF-10, US-HBF-15, US-HBF-20, US-HBF-25, and US-HBF-30. Preliminary experiments showed that when the substitution amount of SG-HBF or US-HBF exceeded 30%, a dough with a strong network structure could not be formed due to insufficient gluten content of the mixed flour. Therefore, the maximum substitution ratio of SG-HBF or US-HBF was set at 30%. The samples without HBF were used as the control group (ck).

### 2.4. Pasting Properties of Mixed Flour

According to the AOAC methods, the moisture content of the flour sample was determined using the 925.10 method by drying at 105 °C until a consistent weight was noted. The pasting properties of the samples were measured by a rapid viscosity analyzer (RVA, RVA 4500, Peten Instruments (Beijing) Co., Ltd., Beijing, China) according to the method described by Han et al. with slight modifications [17]. The 3.00 g sample (14% moisture basis) was accurately weighed into RVA canisters, followed by the addition of 25 mL of distilled water. The determination process was executed as follows: the sample was held at 50 °C for up to 1 min and raised to 95 °C over 3.7 min, then held at 95 °C for 2.7 min, cooled to 50 °C over 3.8 min, and held at 50 °C for 2 min. During the heating process, the sample was stirred by the analyzer at 960 rpm for 10 s, and then at 160 rpm for the rest of the experiment.

### 2.5. Scanning Electron Microscopy

The microstructure of starch samples was observed according to the method described by Fu et al. with slight modifications [18]. A small amount of starch sample was placed on an aluminum carrier table using conductive adhesive tape. The carrier table was then placed in a gold plating instrument, and the samples were coated with carbon for 90 s using an ion sputtering coating instrument. After coating, the samples were examined using a scanning electron microscope (Quanta 200 scanning electron microscope, FEI company, Hillsboro, OR, USA).

### 2.6. X-Ray Diffraction of Mixed Flour

X-ray diffraction patterns of mixed flour samples were determined by using an X-ray diffraction spectrometer (Bruker D8 Advance A25, Bruker AXS SE, Karlsruhe, Germany) according to the method described by Wang et al. with slight modifications [19]. The experimental conditions were set at 30 mA and 40 kV. The diffraction angle (2θ) was scanned from 4° to 40° at a rate of 2°/min. The relative crystallinity of the samples was calculated as the ratio of the crystalline peak area to the total diffraction area.

### 2.7. Preparation of Mixed Dough

The physically modified HBF was mixed with wheat flour (based on 50 g, with substitution ratios of 10%, 15%, 20%, 25%, and 30%). Then water (44%) and salt (1%) were added, and then the mixture was kneaded thoroughly until the dough became soft and smooth, and was allowed to rest for 30 min.

### 2.8. Fourier Transform Infrared Spectroscopy of Dough

FTIR analysis of dough samples was performed according to the method described by Liu et al. with slight modifications [20]. The dough samples were freeze-dried using a vacuum freeze-dryer (Aiphal-2LD plus, Martin Christ Gefriertrocknungsanlagen GmbH, Osterode am Harz, Germany), and then crushed and passed through a 100 mesh sieve. The sample was mixed with potassium bromide (sample: potassium bromide = 1:100, *w*/*w*), ground into fine powder in an agate mortar, and pressed into a pellet for analysis using a Fourier transform infrared spectrometer (Bruker Optics GmbH & Co. KG, Ettlingen, Germany) for scanning. Against the background of potassium bromide, the scanning range was set from 400 to 4000 cm^−1^, with a resolution of 4 cm^−1^ and a scanning frequency of 32 times. Special attention was given to the amide I band within the range of 1600 to 1700 cm^−1^. Using Peakfit v4.12 software, the amide I band spectrum was analyzed by first correcting the baseline and applying Savitsky–Golay function smoothing, followed by Gaussian deconvolution, with a second derivative fit applied to each component. The percentage composition of each secondary structure type is determined by dividing the area of each sub-peak in the respective region by the total integrated area.

### 2.9. Preparation of Noodles

Noodles were prepared according to the method described by Wu et al. with slight modifications [21]. The dough was pressed by a press with a 3 mm gap, folded and pressed 3 times. The dough was placed in a self-sealing bag for 30 min, and then passed through the gaps of 2.50, 2.00, 1.50 and 1.25 mm in turn. Finally, the dough was cut into 2 mm wide and 400 mm long noodles, and sealed with plastic film. The fresh noodles were boiled in 2000 mL boiling water. The cooked noodles were soaked in 500 mL of cold water (20 °C) for 30 s. Then, the noodles were strained using a strainer to remove the water droplets on the surface of the noodles.

#### 2.9.1. Color of Noodles

The color of noodles was determined using a model CR-400 color difference meter (Konica Minolta, Sakai, Osaka, Japan) according to the method described by Zhu et al. with slight modifications [22]. The L* values, a* values, and b* values of the noodles at different positions were measured respectively.

#### 2.9.2. Water Distribution of Noodles

The water distribution of noodles was analyzed using an NMR imaging analyzer (NMI20-040V-I, Suzhou Newmai Analytical Instrument Co., Ltd., Suzhou, China) according to the method previously reported by us with minor modifications [23]. The resonance center frequency was adjusted using an FID test, and the spin relaxation time (T2) was measured using a CPMG pulse sequence. The CPMG test parameters were as follows: dominant frequency = 20 MHz, offset frequency = 628,049.19 Hz, sampling points TD = 40,014, repeated scanning times NS = 4, sampling interval time TW = 2000 ms, half echo number TE = 0.6 ms, echo number NECH = 10,000, temperature = 32 °C.

#### 2.9.3. Optimal Cooking Time of Noodles

The optimal cooking time of noodles was determined according to the method described by Li et al. with slight modifications [24]. Fifteen noodles were put into boiling water. The noodles were boiled for 4 min over low heat. During this time, one noodle was taken out every 10 s and cut with a knife to observe whether the cross section was uniform and translucent. The optimal cooking time was defined as the time when the white core just disappeared.

#### 2.9.4. Cooking Loss Rate of Noodles

The cooking loss rate of noodles was determined according to the method described by Pan et al. with slight modifications [25]. A certain weight of noodle samples was boiled in 1000 mL of distilled water until the optimal cooking time. After washing the noodles with running water for 30 s, they were cooled naturally for 5 min, dried to constant weight at 105 °C, and weighed as m_2_. At the same time, an equal weight of raw noodles was dried to constant weight under the same conditions, and weighed as m_1_.

The cooking loss rate was calculated as follows in Equation (1):

(1) $$\mathrm{Cooking}\;\mathrm{loss}\;\mathrm{rate}\;(\%)\;=\;\frac{m_{1}-m_{2}}{m_{1}}\times 100\%$$

#### 2.9.5. Broken Rate of Noodles

The broken rate of noodles was determined according to the method described by Yang et al. with slight modifications [26]. One hundred noodles were boiled until the optimal cooking time. The number of intact noodles was counted and recorded as N. The calculation formula was as follows in Equation (2):

(2) $$\mathrm{Broken}\;\mathrm{rate}\;(\%)\;=\;\frac{100-N}{100}\times 100\%$$

#### 2.9.6. Texture of Noodles

The texture properties of cooked noodles were measured using a TA-XT plus texture analyzer (Stable Micro Systems, Ltd., Godalming, Surrey, UK) according to the method described by Chang et al. with slight modifications [27]. The noodles were boiled until the optimal cooking time, then cooled and dried with paper to remove excess moisture, and placed on the measuring table in parallel. TPA mode parameters were as follows: the speed before the test was 2.0 mm/s, the speed during the test and after the test was 1.0 mm/s, the trigger force was 5 g, the compression ratio was 75%, and the time interval between two compressions was 1 s.

#### 2.9.7. Sensory Evaluation of Noodles

The cooked noodles were promptly served to the assessors within 5 min after cooking. All noodle samples were coded with random three-digit numbers for blind evaluation and served to assessors in a randomized sequence. The sensory evaluation was conducted by twenty trained assessors (10 males and 10 females), who scored the color, appearance, taste, flavor, hardness, toughness, and overall acceptability of the noodle samples. The scores were recorded using a 9-point hedonic scale (1: extremely disliked; 5: neither liked nor disliked; 9: extremely liked) [28].

### 2.10. Statistical Analysis

All experiments were conducted in triplicate, and results are expressed as mean ± standard deviation (SD). Data analysis was performed using Microsoft Excel, SPSS 26.0 (IBM Corporation, Chicago, IL, USA), and Origin 2022 (Origin Lab Corporation, Northampton, MA, USA). Analysis of variance (ANOVA) and Duncan’s test were used to assess significant differences. Statistical significance was set at *p* < 0.05.

## 3. Results and Discussion

### 3.1. Pasting Properties of Mixed Flour

The effects of different substitution ratios of modified HBF on the pasting properties of flour are shown in Table 1. The peak viscosity is an important indicator of cooked noodle quality, showing significantly positive correlations with stickiness, appearance, and smoothness. Within the substitution range of 10–30%, as the amount of SG-HBF increased, the PV, TV, and FV of the mixed flour decreased. This may be because the structure of HB starch granules was destroyed by SG treatment. Additionally, SG-HBF enhanced the interaction between starch granules, making the gelatinization process easier and thereby reducing the viscosity of the mixed flour. The BD value reflects the shear resistance of starch during heating. The smaller the BD value, the better the pasting stability [29]. The SB value reflects the short-term aging ability of starch [30]. Compared with the ck group, the addition of SG-HBF reduced the BD and SB values of the mixed flour, indicating that SG-HBF improved the pasting stability and anti-aging ability of the mixed flour. With increasing US-HBF substitution, the TV and FV of the mixed flour increased first and then decreased, while the BD and SB increased significantly (*p* < 0.05). This might be because the US treatment destroyed the molecular structure of HB starch, allowing water molecules to more easily enter the interior of macromolecules, thereby increasing the BD and SB values. However, with a higher amount of modified HBF, the dietary fiber in the modified HBF competed with starch molecules for water molecules, inhibiting the intermolecular combinations between starch-starch and starch-protein, thereby causing a significant decrease (*p* < 0.05) in the viscosity of the mixed flour [31,32]. Under the same substitution ratios, the pasting indexes of the mixed flour with US-HBF were significantly higher (*p* < 0.05) than those of SG-HBF.

### 3.2. Microstructure of Mixed Flour

**Figure 1:**
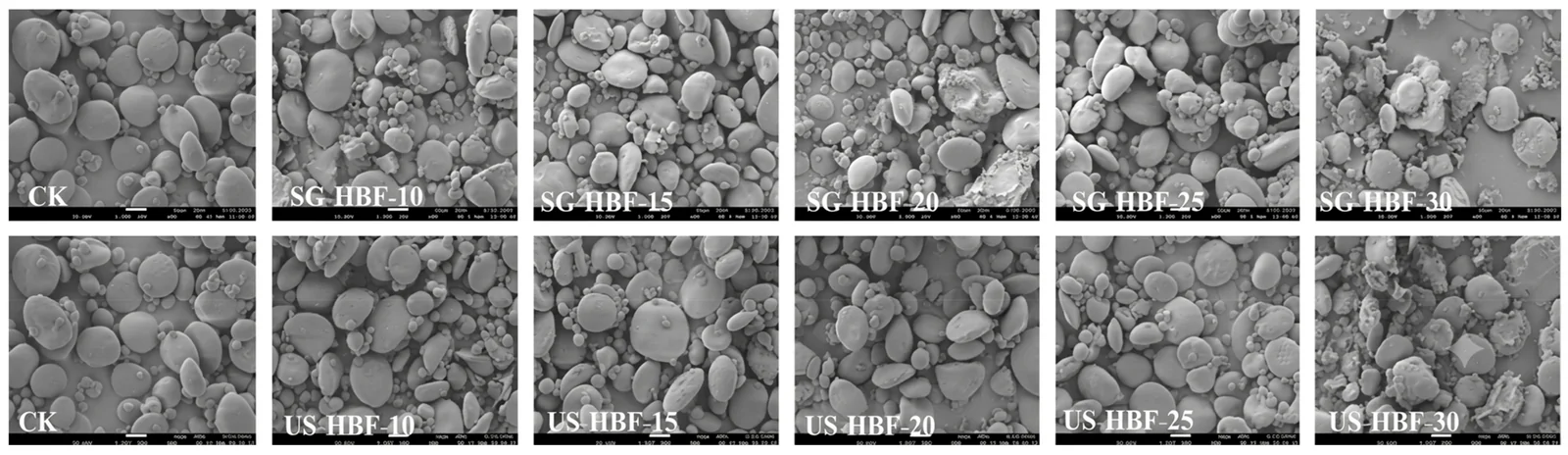
SEM images of flour mixed with different substitution ratios of SG-HBF and US-HBF.

The effects of different substitution ratios of modified HBF on the microstructure properties of mixed flour are shown in Figure 1. As the substitution amount of physically modified HBF increased, the HB starch granules gradually increased in number. When SG-HBF substitution exceeded 15%, the surface of barley starch granules became rough, and the oval or spherical morphology was destroyed to varying degrees. When SG-HBF substitution exceeded 25%, HB starch granules began to aggregate partially and were increasingly adsorbed onto wheat starch granules. This may be due to the high specific surface area and electrostatic effect of SG-treated HBF [33]. In contrast, when US-HBF substitution amount was less than 25%, the morphology of HB starch granules remained relatively complete and unbroken. However, at substitution levels above 25%, the HB starch granules began to accumulate in large quantities and adhered to the wheat starch granules. This may be because the US treatment destroyed the cell structure of HB starch [34]. At the same substitution ratio, compared with SG-HBF, US-HBF exhibited better particle integrity and more uniform dispersion. This is consistent with the results of the properties of mixed flour. The more intact granule morphology of US-HBF explains its higher viscosity parameters, as less damaged granules swell more effectively.

### 3.3. X-Ray Diffraction of Mixed Flour

**Figure 2:**
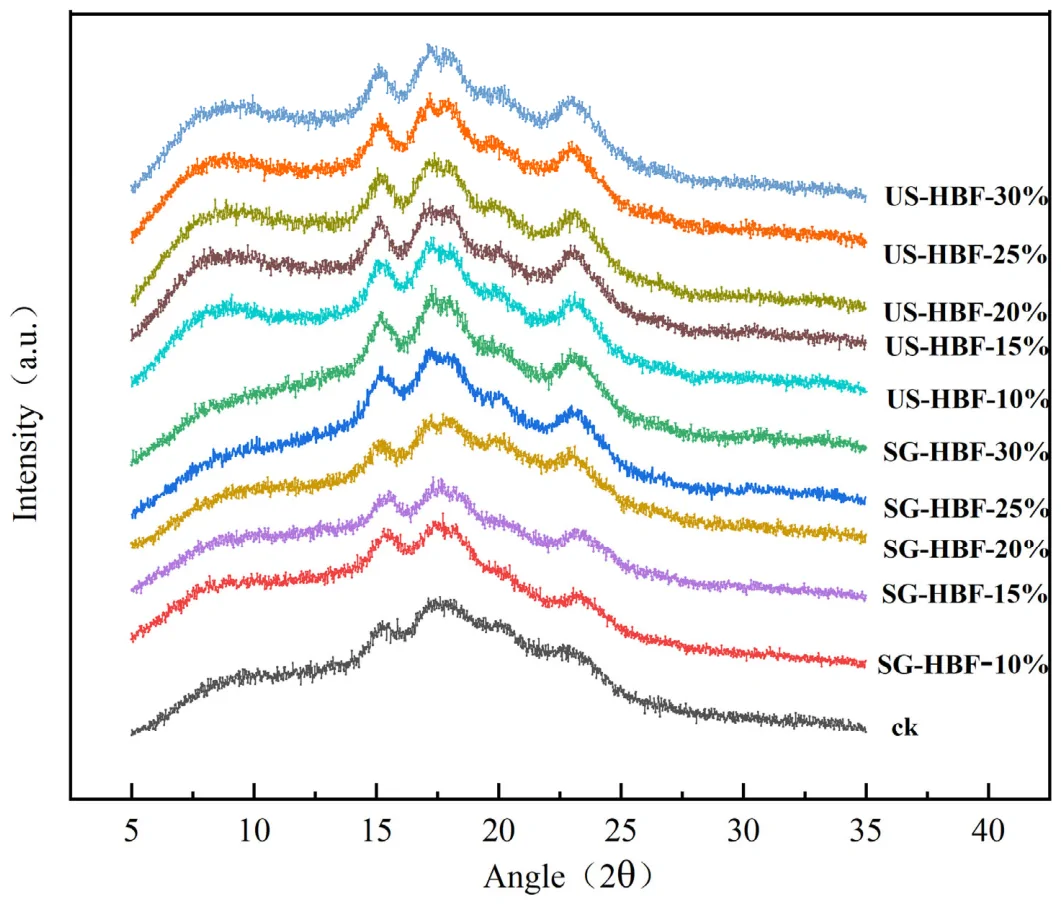
X-ray diffraction patterns of SG-HBF and US-HBF mixed flour.

The X-ray diffraction patterns of mixed flour with different substitution ratios of modified HBF are shown in Figure 2. Neither SG nor US treatment changed the crystal form of wheat flour. All samples exhibited typical A-type X-ray diffraction patterns, with obvious double-shoulder characteristic diffraction peaks at 2θ values of 17° and 18°, and single diffraction peaks at 15° and 23°. The effects of different substitution ratios of modified HBF on relative crystallinity are shown in Table 2. Relative crystallinity refers to the proportion of crystalline regions and reflects the degree of order in the starch structure [35]. With increasing SG-HBF substitution, the relative crystallinity of the mixed flour first increased and then decreased, reaching a maximum value of 70.48% at 20% substitution. The initial increase might be attributed to the preferential disruption of the amorphous regions of starch granules by mechanical forces during SG, which increases the relative proportion of crystalline domains. However, with further increasing SG-HBF substitution (>20%), the cumulative mechanical damage extended to the crystalline regions, leading to progressive disordering and amorphization of the starch crystalline structure, and consequently a decrease in relative crystallinity. The substitution of US-HBF resulted in a relative crystallinity that was generally higher than that of the ck group. This may be because the cavitation effect generated by US first acts on the amorphous region of starch, causing chain scission and subsequent formation of shorter-chain ordered structures, thereby temporarily increasing the crystallinity. However, when the substitution ratio of US-HBF exceeded 15%, the relative crystallinity showed a decreasing trend, indicating that excessive ultrasonic damage began to disrupt the crystalline region. The mechanism differences between the SG and US treatments also explains the differences in the pasting properties of flour and the subsequent texture of the noodles [36].

### 3.4. Fourier Transform Infrared Spectroscopy of Dough

**Figure 3:**
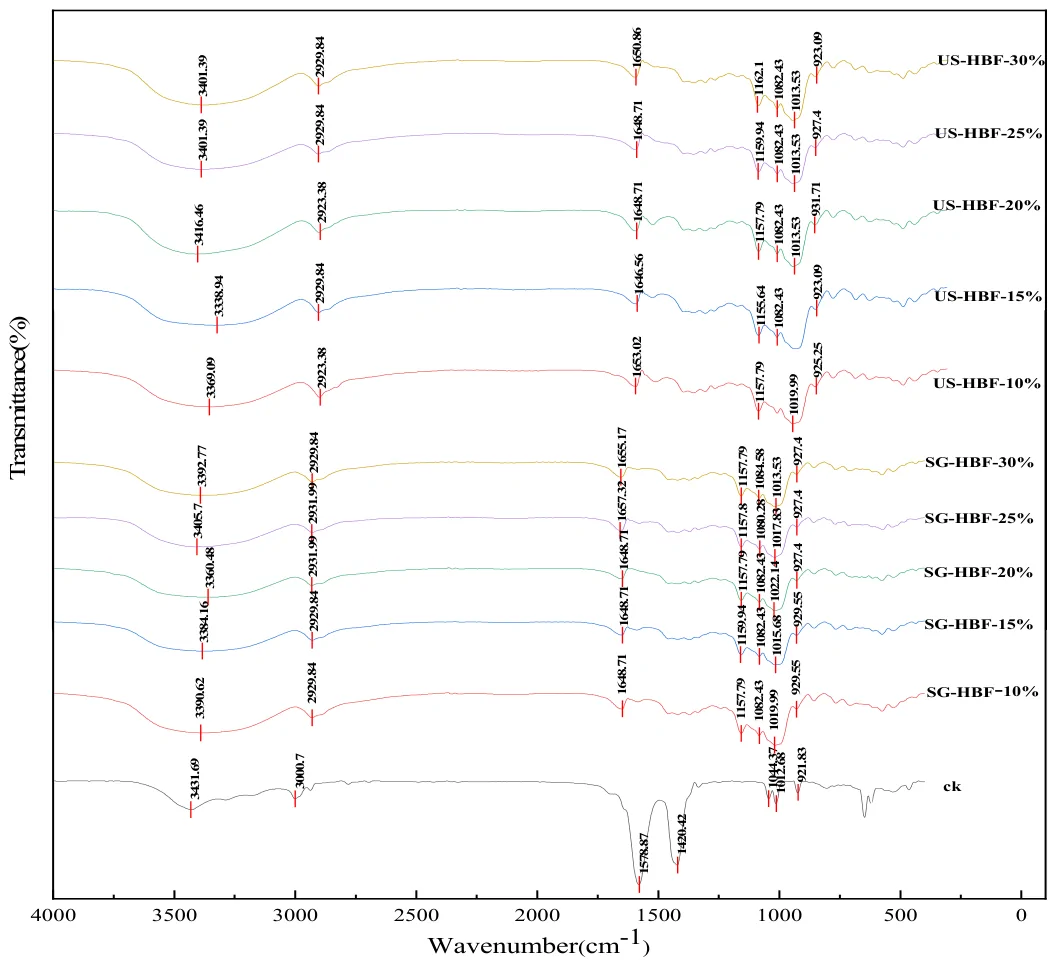
FTIR spectra of dough with different substitution ratios of SG-HBF and US-HBF.

The FTIR spectra of dough with different substitution ratios of modified HBF are shown in Figure 3. The 1200–800 cm^−1^ region spectrum represents C-OH, C-H, C-C stretching vibration and C-O bending vibration. The infrared absorption at 921 cm^−1^, 1044 cm^−1^, and 1157 cm^−1^ are more sensitive to the conformation of the mixed flour, which is attributed to the influence of the physically modified HBF on the mixed flour. In the region of 3700–3100 cm^−1^, there is a strong absorption peak in the dough, likely caused by the -OH stretching vibration. As the proportion of physically modified HBF increased, the absorption peak shifted to the right. Specifically, the peak position for the ck dough was 3431.69 cm^−1^. With increasing SG-HBF substitution ratios, the absorption peak shifted progressively to 3390.62 cm^−1^, 3384.16 cm^−1^, 3360.48 cm^−1^, 3405.70 cm^−1^, and 3392.77 cm^−1^, respectively. For US-HBF doughs, the absorption peak shifted to 3369.09 cm^−1^, 3338.94 cm^−1^, 3416.46 cm^−1^, 3401.39 cm^−1^, and 3401.39 cm^−1^, respectively. This shift to right wavenumbers indicates that the addition of physically modified HBF led to an increase in the hydrogen bond content in the mixed dough. This may be because water molecules can more easily form hydrogen bonds with the functional groups in the polymer [37]. The 1800–1500 cm^−1^ band corresponds to the absorption peak of protein amide I and II bands. Compared with ck group, the intensity of the absorption peak near 1650 cm^−1^ decreased and shifted, indicating that the addition of physically modified HBF altered the secondary structure of protein in the dough system.

To quantitatively evaluate the changes in protein secondary structure induced by the substitution of SG-HBF and US-HBF, the Amide I band (1600–1700 cm^−1^) was subjected to Gaussian deconvolution combined with second-derivative analysis (Table 3). In the gluten network, α-helix and β-sheet are stable secondary protein structures formed by hydrogen bonds, providing elasticity and rigidity to the dough, while β-turn plays a key role in chain reversal and folding of the protein molecule, and random coils confer conformational flexibility [38,39]. Compared with ck group, β-sheet content decreased significantly with increasing substitution ratios of SG-HBF. This reduction might be attributed to the dilution of gluten proteins by non-gluten components from HBF, which interferes with the inter-chain hydrogen bonds and hydrophobic associations. α-helix and random coil contents first increased and then decreased, both reaching maximum values at 15% substitution. The initial increase at low substitution levels suggests a conformational rearrangement of gluten proteins, reflecting the unfolding of polypeptide chains upon SG-HBF addition. However, excessive substitution (>15%) causes the gluten network to be overly damaged and loses its ability to maintain an ordered structure. When the substitution ratio exceeded 15%, β-turn content increased significantly, further confirming the transition of gluten network toward a more disordered conformation. β-sheet content also decreased significantly with increasing substitution ratios of US-HBF, while α-helix content increased. β-Turn and random coil contents first increased and then decreased. Under the same substitution amount, US-HBF induced larger changes in gluten secondary structure fractions than SG-HBF, indicating that US-HBF exerted stronger interference with the gluten network than SG-HBF.

### 3.5. Color of Noodles

**Figure 4:**
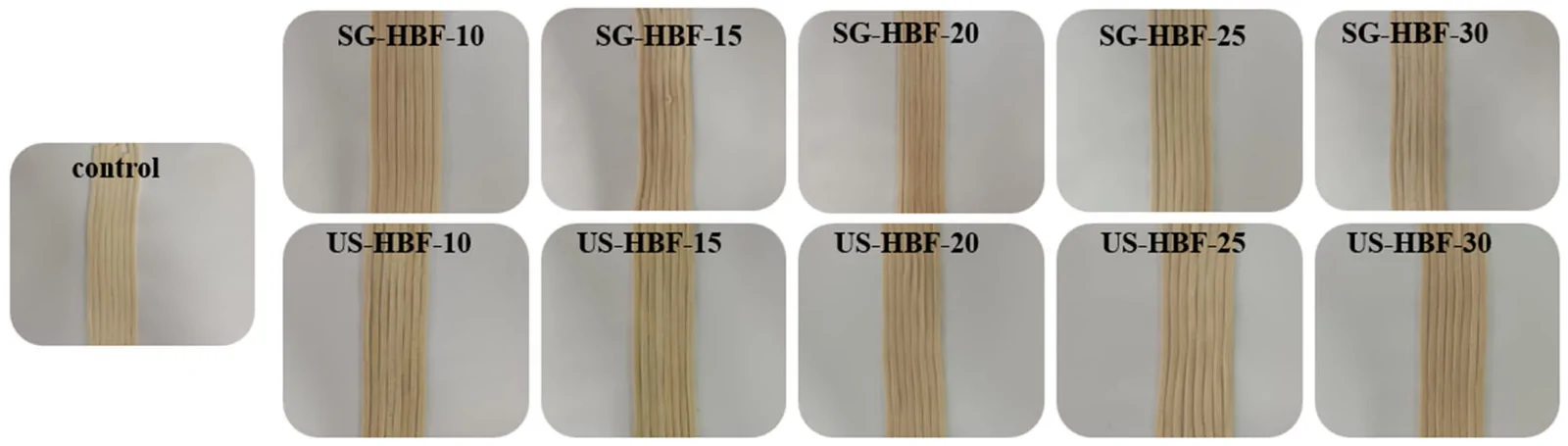
Images of noodles with different substitution ratios of SG-HBF and US-HBF.

The color of noodles has a significant impact on sensory quality and the attractiveness of the noodle product [40]. Since the HBF itself is gray-brown, its addition inevitably changes noodle color. The colors of noodles with different substitution ratios of SG-HBF and US-HBF are shown in Figure 4. The addition of modified HBF reduced the brightness of the noodles and deepened the color. The effects of different substitution ratios of SG-HBF and US-HBF on the color parameters of noodles are shown in Table 4. The L* value (brightness value) and b* value (yellowness value) of the noodles decreased with increasing substitution of physically modified HBF, while the a* value (redness value) increased. This was due to the gray-brown color of the HBF itself. Under the same substitution amount, the L* and a* values of the noodles with SG-HBF were lower than those of the noodles with US-HBF, while the b* values were higher than those of the noodles with US-HBF. This may be because SG treatment produced smaller HBF particle sizes, enabling them to mix more uniformly with the wheat flour, thus achieving better color integration.

### 3.6. Water Distribution of Noodles

The effects of different substitution ratios of modified HBF on the water distribution of noodles are shown in Table 5. For all noodles, two distinct water populations were observed, centered at around 5.93–10.97 ms (T_21_), and 91.12–202 ms (T_22_). T_21_ corresponds to the water that is tightly bound to large molecules such as starch and protein. T_22_ corresponds to free water, and its motility is not bound by gluten proteins. The ratio of each peak area to the total integral area (A_21_ and A_22_) represents the relative percentage content of each water fraction in the noodles [41].Compared with the ck group, the substitution of SG-HBF significantly increased T_21_, with the highest value achieved at a substitution rate of 20%. The increase in T_21_ indicates an enhanced mobility of bound water, which may be attributed to the increased specific surface area of SG-HBF and the increased number of exposed hydrogen bond sites, thereby promoting more extensive interactions between water and macromolecules. However, when the substitution rate of SG-HBF exceeded 20%, T_21_ began to decrease, and the mobility of bound water gradually weakened. For US-HBF, when the substitution ratio was ≤20%, T_21_ remained close to the ck. However, when the substitution ratio reached 30%, T_21_ dropped sharply. This may be due to the destructive effect of US on the gluten network and the competitive hydration effect between dietary fiber and gluten proteins. The observed shorter T_21_ relaxation time at high US-HBF substitution ratios indicates that although water molecules may be more closely bound to the matrix, the overall network structure is disrupted, resulting in limited effective water distribution in the noodles. Regarding the relative content of bound water (A_21_), when the SG-HBF substitution ratio was ≤20%, A_21_ remained at a relatively high level, indicating that the noodles had good water retention ability. When the substitution ratio of SG-HBF was ≥25%, A_21_ significantly decreased and the free water content (A_22_) significantly increased, which was consistent with the observed aggregation of HB starch granules in the SEM. For US-HBF, compared with the ck group, at substitution ratios of 10–30%, A_21_ significantly decreased and A_22_ significantly increased. This may be attributed to the severe structural damage caused by US treatment, which negatively affects the water absorption and network formation of gluten. The observed T_22_ relaxation time was shorter in both noodles at high substitution levels, indicating stronger water retention ability but a less effective distribution, likely due to the competition between dietary fibers and gluten proteins for limited water resources in the noodles matrix [42].

### 3.7. Cooking Characteristics of Noodles

**Figure 5:**
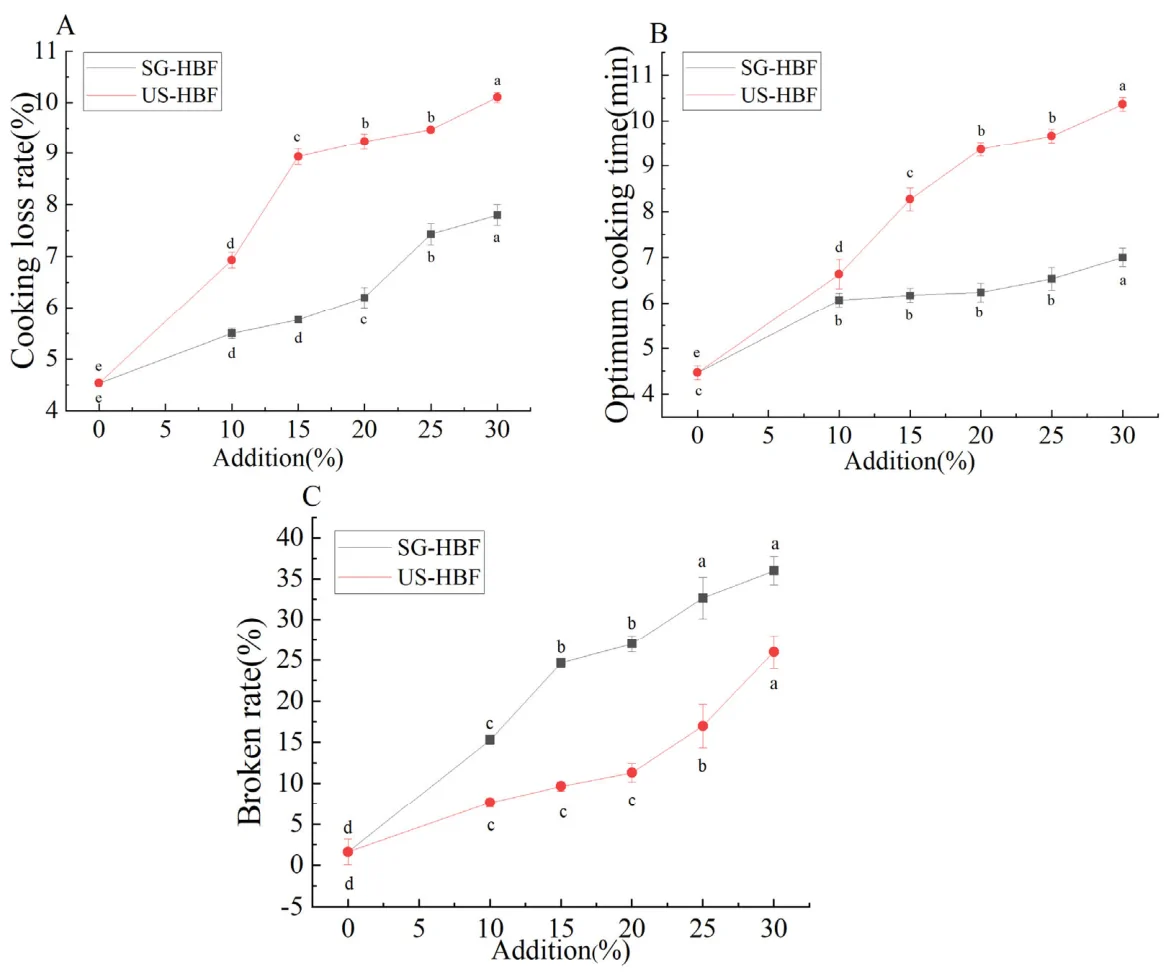
The effects of different substitution ratios of modified HBF on the cooking characteristics of noodles ((**A**): Cooking loss rate; (**B**): Optimum cooking time; (**C**): Broken rate). a–d: This signifies substantial disparities among several letters within the same column of samples (*p* < 0.05).

The effects of different substitution ratios of modified HBF on the noodles cooking characteristics are shown in Figure 5. Three key parameters were evaluated, including the cooking loss rate (Figure 5A), optimal cooking time (Figure 5B), and broken rate (Figure 5C). With increasing substitution ratios of physically modified HBF, all three parameters increased significantly (*p* < 0.05). Compared with the ck group, when the substitution rate of SG-HBF was less than 20%, the cooking loss rate of noodles increased slowly, but increased sharply when the substitution ratio exceeded 20%. This might be due to the SG treatment reducing the particle size, resulting in an increase in specific surface area, enhanced water absorption capacity, but also weakening the interaction between gluten proteins and starch particles. When the SG-HBF substitution ratio exceeded 20%, the cumulative structural damage to the gluten network reached a critical level, leading to a sharp rise in cooking loss. As the SG-HBF substitution ratio increased, the optimal cooking time increased, but remained stable within the range of 10–30%, indicating good processing stability. For US-HBF noodles, when the substitution rate of US -HBF was less than 15%, the cooking loss rate increased sharply, and the rate of increase slowed down beyond 15%. US treatment caused severe disruption of starch and gluten structures, resulting in a weaker and less continuous noodle matrix, which allowed more starch and soluble solids to leach into the boiling water. Moreover, the increased proportion of free water in US-HBF noodles may facilitate the migration and loss of water-soluble components during boiling. The optimal cooking time increased rapidly within the substitution range of 0–20%, and the rate of increase slowed down beyond 20%. Under the same substitution ratios, the cooking loss rate and optimal cooking time of US-HBF noodles were higher than those of SG-HBF noodles; whereas, the broken rate was lower. The US treated flour exhibited a more compact structure and enhanced water-holding capacity, requiring more time for heat and water to penetrate the noodle interior, thus providing a longer cooking time and better resistance to breakage [43].

### 3.8. Texture Properties of Noodles

The effects of different substitution ratios of modified HBF on the textural properties of noodles are shown in Table 6. Compared with the ck group, with increasing substitution ratios of physically modified HBF, the hardness and chewiness of the noodles improved significantly (*p* < 0.05). This is mainly attributed to the high dietary fiber content in the HB flour. Dietary fiber can absorb water and expand, increasing the density and compactness of the noodle base, thus making the noodles less prone to deformation under stress. Moreover, the addition of HBF reduced the formation of gluten proteins, further increasing the hardness and chewiness of the noodles. This result is consistent with the study of Deng et al., who reported that the addition of modified potato starch increased the hardness or chewiness of the dough and noodles [44]. The substitution of modified HBF reduced the cohesiveness and resilience of noodles. As gluten content decreases and dietary fiber competes for water, gluten hydration becomes insufficient, leading to a less cohesive and less recoverable noodle structure. This effect was more pronounced for US-HBF noodles, indicating that US-HBF exerted stronger interference with the gluten network than SG-HBF, consistent with the changes in protein secondary structure observed by FTIR. Notably, since the US treatment was applied only to HBF, the observed weakening of the dough network is driven by the wheat gluten dilution and the water competitive by barley β-glucans and damaged starch, not by direct cavitation cleavage of wheat gluten. Modified HBF could actively regulate the elasticity of noodles by enhancing water retention and network integrity. However, excessive substitution (SG-HBF > 25%, US-HBF > 20%) leads to gluten dilution and network disruption, resulting in a decrease in the springiness of the noodles.

### 3.9. Sensory Score of Noodles

**Figure 6:**
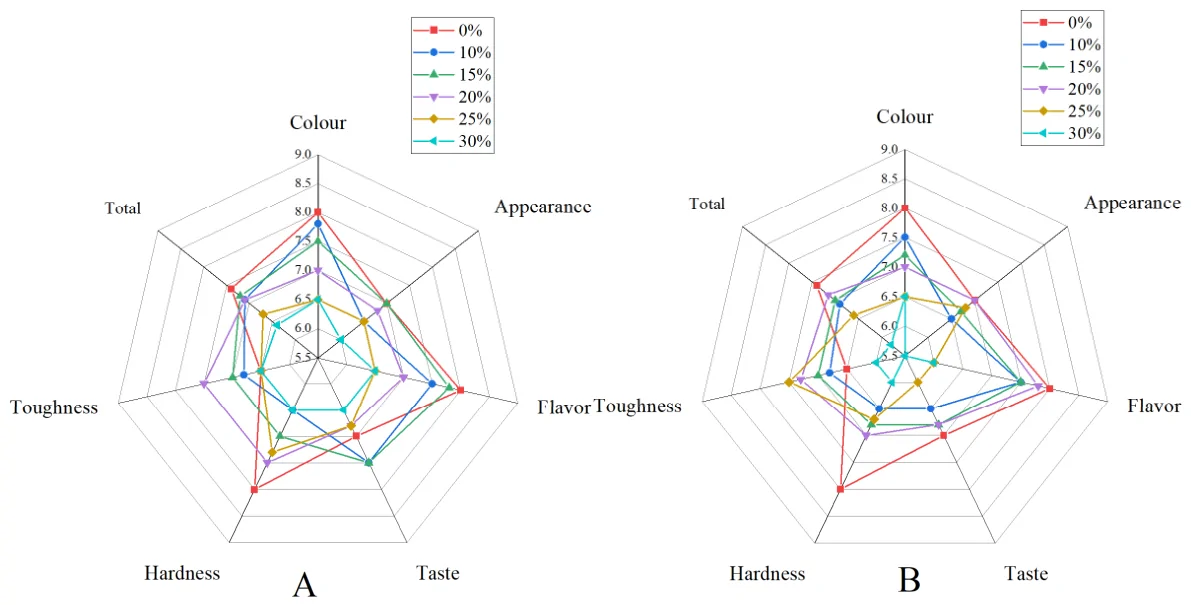
Changes in sensory evaluation of noodles under different substitution ratios of SG-HBF (**A**) and US-HBF (**B**).

Changes in sensory evaluation of wheat flour noodles as a function of different substitution ratios of modified HBF are shown in Figure 6. The sensory scores of noodles decreased progressively with increasing substitution of modified HBF. As the substitution ratios of modified HBF increased, the sensory evaluation indicators for cooked noodles showed a downward trend. When the substitution ratio of modified HBF was less than 20%, all sensory indicators remained at an acceptable level (above 7 points). When it exceeded 20%, the noodle scores were lower and the sensory quality was unacceptable. The high content of HBF introduced a bitter, bran-like taste, masking the wheat aroma, resulting in a decline in flavor and taste. The higher dietary fiber content of HBF also led to an increase in noodle hardness and a deterioration in taste. The decrease in the color score of the noodles was mainly due to the gray-brown color of HBF itself. At the same substitution rate, the sensory score of SG-HBF noodles was higher than that of US-HBF noodles, which was consistent with their lower cooking loss, shorter cooking time, and better color integration.

## 4. Conclusions

This study systematically investigated and compared the effects of substituting wheat flour with SG-HBF and US-HBF on the properties of wheat flour, dough and noodles. Within the substitution range of 10–30%, SG-HBF reduced peak viscosity, breakdown, and setback, indicating improved thermal stability and anti-retrogradation properties; whereas, US-HBF significantly increased the viscosity of the mixed flour. SG-HBF or US-HBF decreased the brightness and deepened the color of the noodles. With increasing substitution ratios of modified HBF, the gluten network transforms from an ordered structure to disordered conformation. Water distribution analysis showed that the bound water content remained relatively high when the substitution ratio of SG-HBF was less than 20% or that of US-HBF was less than 10%. When the substitution ratio of SG-HBF was less than 15% and the substitution ratio of US-HBF was less than 20%, they had positive effects on the microstructure and starch crystalline properties, and the noodles could maintain good sensory and cooking quality. Excessive SG-HBF (>15%) or US-HBF (>20%) increased the cooking loss rate of noodles, reduced the springiness of noodles, and lowered the sensory quality of noodles. SG-HBF offers lower cooking loss and shorter cooking time, suitable for processing efficiency. US-HBF provides higher springiness and lower broken rate, making it suitable for products requiring noodle integrity. The results of this study establish the application value of SG-HBF and US-HBF in noodles, and provide a theoretical basis for the in-depth application of physically modified HBF in wheat products.

## Figures and Tables

**Table 1:** Effects of SG-HBF and US-HBF on pasting properties of flour.

Samples	PV (cP)	TV (cP)	BD (cP)	FV (cP)	SB (cP)
ck	1887.00 ± 83.02 ^a^	1294.33 ± 66.94 ^abcd^	459.33 ± 6.11 ^b^	2378.67 ± 44.74 ^bcd^	1084.33 ± 23.09 ^b^
SG-HBF-10	1722.67 ± 19.14 ^ab^	1271.00 ± 33.15 ^bcd^	371.00 ± 44.03 ^b^	2313.33 ± 78.78 ^cd^	943.33 ± 54.10 ^d^
SG-HBF-15	1630.00 ± 17.52 ^b^	1218.67 ± 71.29 ^cd^	402.33 ± 4.93 ^b^	2262.67 ± 71.34 ^d^	967.67 ± 41.68 ^cd^
SG-HBF-20	1612.00 ± 57.47 ^b^	1244.33 ± 34.65 ^bcd^	404.33 ± 18.0 ^b^	2224.00 ± 162.53 ^d^	1013.00 ± 93.05 ^bc^
SG-HBF-25	1572.33 ± 89.40 ^b^	1236.67 ± 62.01 ^bcd^	431.33 ± 8.74 ^b^	2204.33 ± 46.49 ^d^	1044.00 ± 13.53 ^bc^
SG-HBF-30	1577.67 ± 67.89 ^b^	1206.67 ± 49.86 ^abc^	451.67 ± 19.14 ^b^	2183.33 ± 47.83 ^d^	1075.67 ± 3.06 ^b^
US-HBF-10	1875.67 ± 36.86 ^a^	1433.00 ± 55.02 ^a^	649.33 ± 10.90 ^a^	2621.67 ± 33.98 ^a^	1233.33 ± 47.93 ^a^
US-HBF-15	1884.33 ± 88.99 ^a^	1346.67 ± 11.02 ^ab^	652.67 ± 42.47 ^a^	2595.67 ± 34.08 ^ab^	1238.67 ± 35.10 ^a^
US-HBF-20	1849.67 ± 77.78 ^a^	1318.00 ± 105.43 ^ab^	665.00 ± 34.51 ^a^	2615.67 ± 213.20 ^a^	1240.67 ± 33.31 ^a^
US-HBF-25	1839.00 ± 59.92 ^a^	1253.00 ± 72.99 ^bc^	671.00 ± 40.58 ^a^	2491.67 ± 99.42 ^abc^	1250.00 ± 26.46 ^a^
US-HBF-30	1834.00 ± 62.69 ^a^	1148.83 ± 128.17 ^d^	680.67 ± 55.36 ^a^	2391.33 ± 186.83 ^cd^	1255.33 ± 38.51 ^a^

PV (Peak viscosity), TV (Trough viscosity), BD (Breakdown), FV (Final Viscosity), SB (Setback). a–d: This signifies substantial disparities among several letters within the same column of samples (*p* < 0.05). The values are shown as the mean ± standard deviation.

**Table 2:** Effects of SG-HBF and US-HBF on the relative crystallinity of mixed flour.

Samples	Relative Crystallinity (%)
ck	66.78 ± 0.05 ^f^
SG-HBF-10	67.64 ± 0.08 ^e^
SG-HBF-15	68.18 ± 0.01 ^d^
SG-HBF-20	70.48 ± 0.01 ^a^
SG-HBF-25	66.11 ± 0.01 ^g^
SG-HBF-30	64.52 ± 0.06 ^h^
US-HBF-10	69.91 ± 0.06 ^b^
US-HBF-15	69.54 ± 0.10 ^b^
US-HBF-20	68.97 ± 0.55 ^c^
US-HBF-25	68.82 ± 0.04 ^c^
US-HBF-30	68.22 ± 0.68 ^d^

a–h: This signifies substantial disparities among several letters within the same column of samples (*p* < 0.05). The values are shown as the mean ± standard deviation.

**Table 3:** Effects of SG-HBF and US-HBF on the secondary structure of dough.

Samples	β-sheet (%)	α-helix (%)	β-turn (%)	Random coil (%)
ck	35.14 ± 0.27 ^a^	12.21 ± 0.23 ^i^	40.69 ± 0.20 ^f^	11.96 ± 0.24 ^g^
SG-HBF-10	27.92 ± 0.39 ^b^	16.32 ± 0.35 ^h^	36.11 ± 0.39 ^g^	19.65 ± 0.34 ^e^
SG-HBF-15	21.16 ± 0.19 ^d^	20.47 ± 0.34 ^e^	34.92 ± 0.19 ^h^	23.44 ± 0.35 ^c^
SG-HBF-20	23.49 ± 0.39 ^c^	16.38 ± 0.19 ^h^	41.94 ± 0.39 ^e^	18.19 ± 0.19 ^f^
SG-HBF-25	21.17 ± 0.34 ^d^	17.75 ± 0.31 ^g^	43.03 ± 0.38 ^d^	18.03 ± 0.26 ^f^
SG-HBF-30	15.28 ± 0.29 ^f^	18.68 ± 0.17 ^f^	47.80 ± 0.28 ^b^	18.23 ± 0.18 ^f^
US-HBF-10	8.07 ± 0.27 ^i^	21.56 ± 0.16 ^d^	48.94 ± 0.23 ^a^	21.42 ± 0.17 ^d^
US-HBF-15	9.89 ± 0.22 ^h^	22.84 ± 0.29 ^c^	42.03 ± 0.39 ^e^	25.24 ± 0.18 ^b^
US-HBF-20	19.30 ± 0.19 ^e^	22.91 ± 0.25 ^c^	32.75 ± 0.25 ^i^	25.03 ± 0.16 ^b^
US-HBF-25	11.92 ± 0.26 ^g^	25.24 ± 0.34 ^a^	32.26 ± 0.34 ^i^	30.58 ± 0.26 ^a^
US-HBF-30	7.79 ± 0.42 ^i^	24.49 ± 0.39 ^b^	43.97 ± 0.38 ^c^	23.74 ± 0.51 ^c^

a–i: This signifies substantial disparities among several letters within the same column of samples (*p* < 0.05). The values are shown as the mean ± standard deviation.

**Table 4:** Effects of different substitution ratios of SG-HBF and US-HBF on the color parameters of noodles.

Samples	L*	a*	b*
ck	85.94 ± 1.03 ^a^	−1.27 ± 0.06 ^e^	18.78 ± 0.24 ^ab^
SG-HBF-10	82.76 ± 0.26 ^b^	0.47 ± 0.16 ^d^	16.91 ± 0.59 ^c^
SG-HBF-15	78.40 ± 0.70 ^de^	1.26 ± 0.27 ^c^	17.94 ± 0.64 ^bc^
SG-HBF-20	78.37 ± 1.83 ^de^	1.66 ± 0.22 ^b^	19.02 ± 0.70 ^ab^
SG-HBF-25	77.95 ± 0.57 ^de^	1.72 ± 0.13 ^b^	18.23 ± 0.19 ^ab^
SG-HBF-30	75.87 ± 0.24 ^e^	2.02 ± 0.08 ^a^	18.26 ± 0.23 ^a^
US-HBF-10	83.11 ± 1.72 ^b^	0.71 ± 0.13 ^d^	15.51 ± 0.92 ^d^
US-HBF-15	82.27 ± 0.24 ^b^	1.28 ± 0.09 ^c^	15.34 ± 0.14 ^de^
US-HBF-20	81.40 ± 0.66 ^bc^	1.74 ± 0.07 ^ab^	15.23 ± 0.30 ^def^
US-HBF-25	79.70 ± 1.18 ^cd^	2.03 ± 0.12 ^a^	14.04 ± 0.77 ^ef^
US-HBF-30	76.94 ± 1.50 ^de^	2.24 ± 0.08 ^ab^	14.15 ± 0.76 ^f^

a–f: This signifies substantial disparities among several letters within the same column of samples (*p* < 0.05). The values are shown as the mean ± standard deviation.

**Table 5:** Effects of SG-HBF and US-HBF on water distribution in dough.

Samples	T_21_/ms	T_22_/ms	A_21_/%	A_22_/%
ck	7.46 ± 0.63 ^a^	106.65 ± 0.53 ^a^	99.53 ± 0.03 ^a^	0.47 ± 0.03 ^fg^
SG-HBF-10	9.10 ± 0.86 ^b^	172.87 ± 7.30 ^c^	99.57 ± 0.06 ^a^	0.43 ± 0.06 ^g^
SG-HBF-15	9.32 ± 0.00 ^b^	174.75 ± 1.00 ^c^	99.28 ± 0.07 ^bcd^	0.72 ± 0.08 ^de^
SG-HBF-20	10.97 ± 0.00 ^c^	202.32 ± 3.21 ^b^	99.40 ± 0.02 ^b^	0.60 ± 0.02 ^ef^
SG-HBF-25	9.88 ± 0.95 ^ab^	182.22 ± 5.47 ^b^	99.25 ± 0.06 ^cd^	0.74 ± 0.06 ^de^
SG-HBF-30	9.06 ± 0.74 ^b^	148.50 ± 0.00 ^d^	99.11 ± 0.06 ^e^	0.89 ± 0.06 ^bc^
US-HBF-10	7.03 ± 0.03 ^a^	107.23 ± 0.00 ^b^	99.36 ± 0.03 ^bc^	0.64 ± 0.03 ^de^
US-HBF-15	7.53 ± 0.45 ^a^	125.85 ± 0.58 ^a^	99.23 ± 0.04 ^d^	0.77 ± 0.04 ^cd^
US-HBF-20	7.69 ± 1.13 ^a^	114.39 ± 10.30 ^b^	99.00 ± 0.08 ^ef^	1.00 ± 0.08 ^b^
US-HBF-25	7.00 ± 0.03 ^a^	113.07 ± 10.11 ^b^	98.98 ± 0.09 ^f^	1.02 ± 0.16 ^b^
US-HBF-30	5.93 ± 0.02 ^b^	91.12 ± 0.01 ^c^	98.78 ± 0.02 ^g^	1.19 ± 0.06 ^a^

a–g: This signifies substantial disparities among several letters within the same column of samples (*p* < 0.05). The values are shown as the mean ± standard deviation.

**Table 6:** The effects of different substitution ratios of modified HBF on the textural properties of noodles.

Samples	Hardness (g)	Springiness (mm)	Cohesiveness	Chewiness (g⋅mm)	Resilience
ck	8606.17 ± 461.99 ^c^	0.72 ± 0.06 ^d^	0.72 ± 0.01 ^ab^	5333.16 ± 452.26 ^c^	0.42 ± 0.01 ^a^
SG-HBF-10	9744.59 ± 271.64 ^a^	0.73 ± 0.05 ^d^	0.75 ± 0.02 ^a^	5570.49 ± 919.35 ^c^	0.41 ± 0.00 ^a^
SG-HBF-15	9668.22 ± 621.31 ^a^	0.74 ± 0.06 ^cd^	0.74 ± 0.02 ^a^	6064.44 ± 126.59 ^ab^	0.40 ± 0.02 ^ab^
SG-HBF-20	9537.54 ± 376.49 ^ab^	0.75 ± 0.05 ^bcd^	0.69 ± 0.02 ^bc^	7708.16 ± 699.06 ^a^	0.42 ± 0.01 ^a^
SG-HBF-25	9645.33 ± 458.73 ^a^	0.77 ± 0.01 ^abcd^	0.68 ± 0.01 ^bc^	7105.47 ± 733.41 ^a^	0.38 ± 0.02 ^b^
SG-HBF-30	9876.34 ± 386.43 ^a^	0.63 ± 0.02 ^e^	0.69 ± 0.02 ^bc^	7495.14 ± 812.90 ^a^	0.38 ± 0.02 ^b^
US-HBF-10	9788.60 ± 851.87 ^a^	0.82 ± 0.04 ^abc^	0.73 ± 0.04 ^ab^	5846.76 ± 365.06 ^bc^	0.31 ± 0.01 ^c^
US-HBF-15	9646.29 ± 433.91 ^a^	0.83 ± 0.03 ^ab^	0.66 ± 0.01 ^cd^	5903.65 ± 208.75 ^bc^	0.31 ± 0.01 ^c^
US-HBF-20	9599.45 ± 337.96 ^c^	0.84 ± 0.03 ^a^	0.66 ± 0.03 ^cd^	7500.06 ± 33.66 ^a^	0.33 ± 0.01 ^c^
US-HBF-25	9656.11 ± 158.78 ^bc^	0.78 ± 0.04 ^abcd^	0.64 ± 0.00 ^cd^	7032.30 ± 566.66 ^a^	0.32 ± 0.01 ^c^
US-HBF-30	9871.06 ± 271.46 ^a^	0.77 ± 0.03 ^abcd^	0.62 ± 0.00 ^d^	6841.18 ± 158.32 ^ab^	0.32 ± 0.00 ^c^

a–e: This signifies substantial disparities among several letters within the same column of samples (*p* < 0.05). The values are shown as the mean ± standard deviation.

## Data Availability

The original contributions presented in the study are included in the article; further enquiries can be directed to the corresponding author.
